# Quantifying the Effect of Anthropogenic Climate Change on Calcifying Plankton

**DOI:** 10.1038/s41598-020-58501-w

**Published:** 2020-01-31

**Authors:** Lyndsey Fox, Stephen Stukins, Thomas Hill, C. Giles Miller

**Affiliations:** 1 The Natural History Museum, Cromwell Road, London, SW7 5BD UK; 2 Department of Geography, Geology, and Environment, Kingston University London, Penrhyn Road, Kingston upon Thames, Surrey, KT1 2EE UK

**Keywords:** Climate sciences, Ocean sciences

## Abstract

Widely regarded as an imminent threat to our oceans, ocean acidification has been documented in all oceanic basins. Projected changes in seawater chemistry will have catastrophic biotic effects due to ocean acidification hindering biogenic carbonate production, which will in turn lead to substantial changes in marine ecosystems. However, previous attempts to quantify the effect of acidification on planktonic calcifying organisms has relied on laboratory based studies with substantial methodological limitations. This has been overcome by comparing historic plankton tows from the seminal HMS *Challenger* Expedition (1872–1876) with the recent *Tara* Oceans expedition material (2009–2016). Nano CT-scans of selected equatorial Pacific Ocean planktonic foraminifera, have revealed that all modern specimens had up to 76% thinner shells than their historic counterparts. The “*Challenger* Revisited” project highlights the potential of historic ocean collections as a tool to investigate ocean acidification since the early Industrial Revolution. Further analyses of such biotic archives will enable researchers to quantify the effects of anthropogenic climate change across the globe.

## Introduction

The HMS *Challenger* expedition from 1872 to 1876 can claim to be the foundation of modern oceanographic studies. This historic voyage was the first to specifically gather data on a broad range of ocean features, including ocean temperatures, seawater chemistry, currents, marine life, and the geology of the seafloor^1,2^. In 2022 scientists will celebrate 150 years since the HMS *Challenger* first left port to begin this ground-breaking oceanographic expedition. During this time scientific and technological methods have advanced substantially, but the wealth of knowledge associated with the modern ocean systems is in stark contrast to the fundamental lack of baseline ocean data from the Industrial Revolution onwards^3^.

The wealth of historical collections housed at museums and similar institutions across the world can often be overlooked for cutting edge climate research. At the Natural History Museum, London, the Ocean Bottom Deposits (OBD) collection^4^, which includes vast amounts of material from the HMS *Challenger* expedition, provides an almost unique source of microfauna to compare pre-industrial oceans to those of today. In this study: “The *Challenger* Revisited Project”, the collection provides a unique opportunity to study the effects of one of the most urgent questions of our time with regards to anthropogenic environmental change: ocean acidification (OA)^5^.

It is now widely accepted that OA is an imminent threat to our oceans^5,6^, and although we have a good understanding of the related changes in ocean chemistry, the widespread biological impacts of OA remain unclear. Since the beginning of the industrial revolution (1760 onwards), CO_2_ emissions from the burning of fossil fuels and changes in land use have led to an increase in atmospheric CO_2_ levels concentrations of 280 ppm to, presently, over 400 ppm^7^, with a dramatic change in magnitude and rate of the human imprint from 1950 onwards known as “The Great Acceleration”^8^. Without significant mitigation, CO_2_ values are expected to rise to between 550 and 1000 ppm, depending on emission scenarios, by the end of this century^7^.

Oceanic carbonate ion concentrations decrease as a consequence of increased atmospheric CO_2_ levels^9^, which, in turn, has a negative effect on the capacity for calcifying organisms (such as molluscs, crustaceans, corals, and foraminifera) to form their essential skeletal or shell material out of calcium carbonate^9–13^. Worryingly, recent laboratory experiments and marine chemical models suggest that eventually calcification rates could slow to the point where they are outpaced by dissolution, and during the 21st century, corals and other calcifying organisms may suffer a great decline. Planktonic foraminifera are one such group that are highly important contributors to carbon cycling in the ocean^14^. They are not only responsible for a quarter or more of global carbonate production^15^, but also an integral component of the marine food chain^16^. However, to date there is little information available with regards the impact of OA on calcification rates of planktonic foraminifera. While lab-based studies are invaluable in assessing short-term controls on calcification in certain species, they do not allow for adaptation in planktonic foraminifera, which have to date, never reproduced in a laboratory environment^16^.

Attempts to evaluate the impact of OA on calcification in the oceans have been hindered by an inability to directly compare the calcification capability of today’s plankton species with equivalent specimens from exclusively pre-and early-industrial times^17^. Field-based studies have tried to compare the calcification ability of a modern plankton species today with their pre-industrial counterparts by using seafloor sediments to represent ‘pre-industrial’ times. However, one centimeter depth of deep sea sediment may represent 100’s of years of Earth’s history^18^, therefore this method inevitably incorporates a mix of specimens from a large window of geological time, leading to potential circularity when comparing data from these sediments to modern sediment traps or plankton tows.

**Figure 1:**
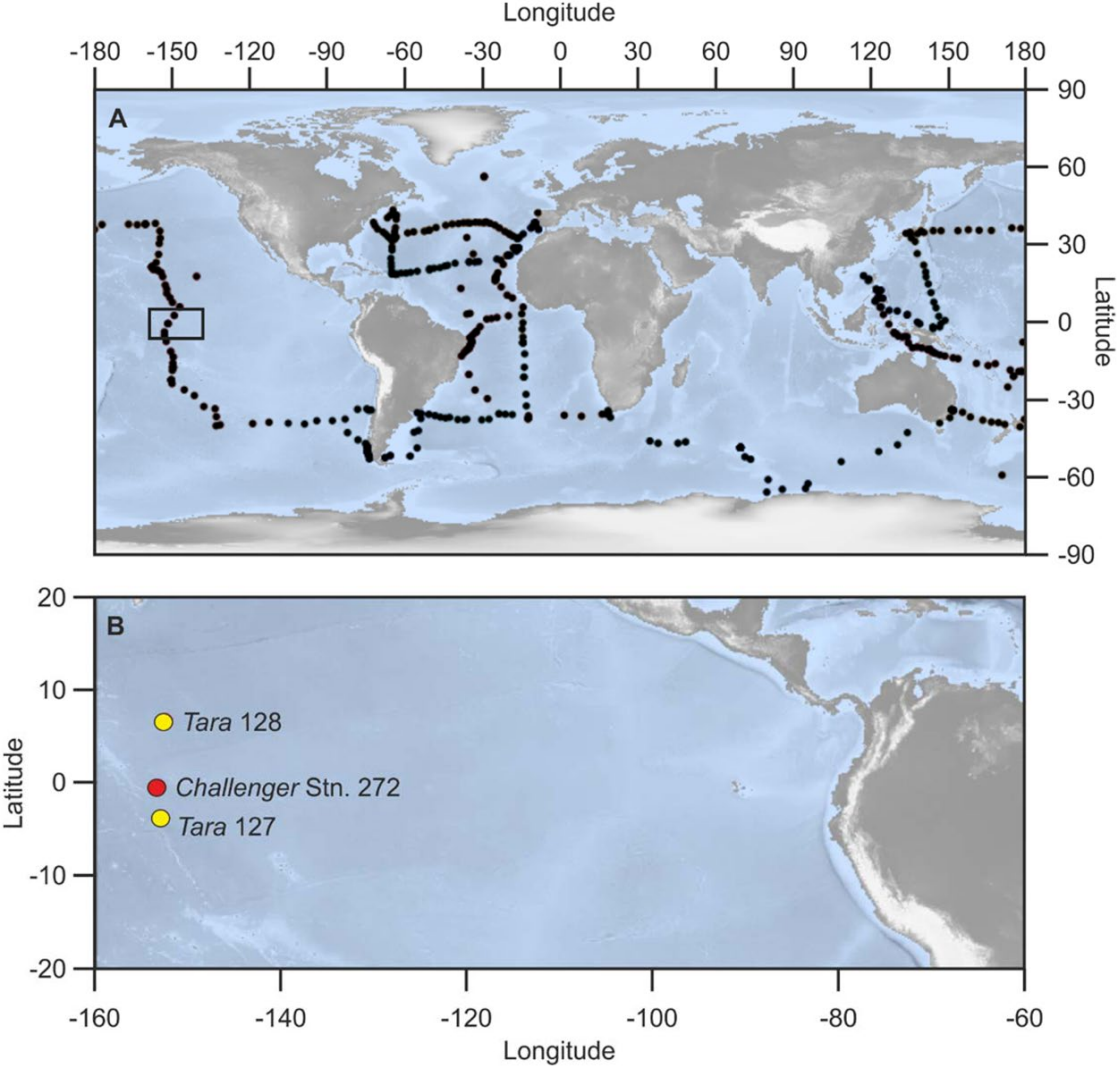
(**A**) Locations of all *Challenger* stations visited between 1872–1876; (**B**) Location map of *Challenger* station 272 and *Tara* stations 127 and 128 used in this study, equatorial Pacific Ocean. Map was generated using the marmap package in R^42^.

Multiple plankton tows collected by the HMS *Challenger* crew from across the world’s ocean basins between 1872–1876^1^ provides a resource that resolves issues highlighted in both lab-based and field-based studies; the tow material contains the planktonic foraminifera, of a known age, that were alive in the open ocean at the time of sampling. Thus, the HMS *Challenger* collection provides exceptional baseline data for 19th–21st century ocean acidification evaluation. Here we present the results of a focused study from the central Pacific Ocean (Fig. 1), a region identified as being vulnerable to ocean warming, ocean acidification, and ocean deoxygenation with increasing atmospheric CO_2_ levels^19,20^. The results of this study highlight the utility of museum collections for studies of anthropogenic climate change, and demonstrates an urgent need for more empirical data.

## Results

**Figure 2:**
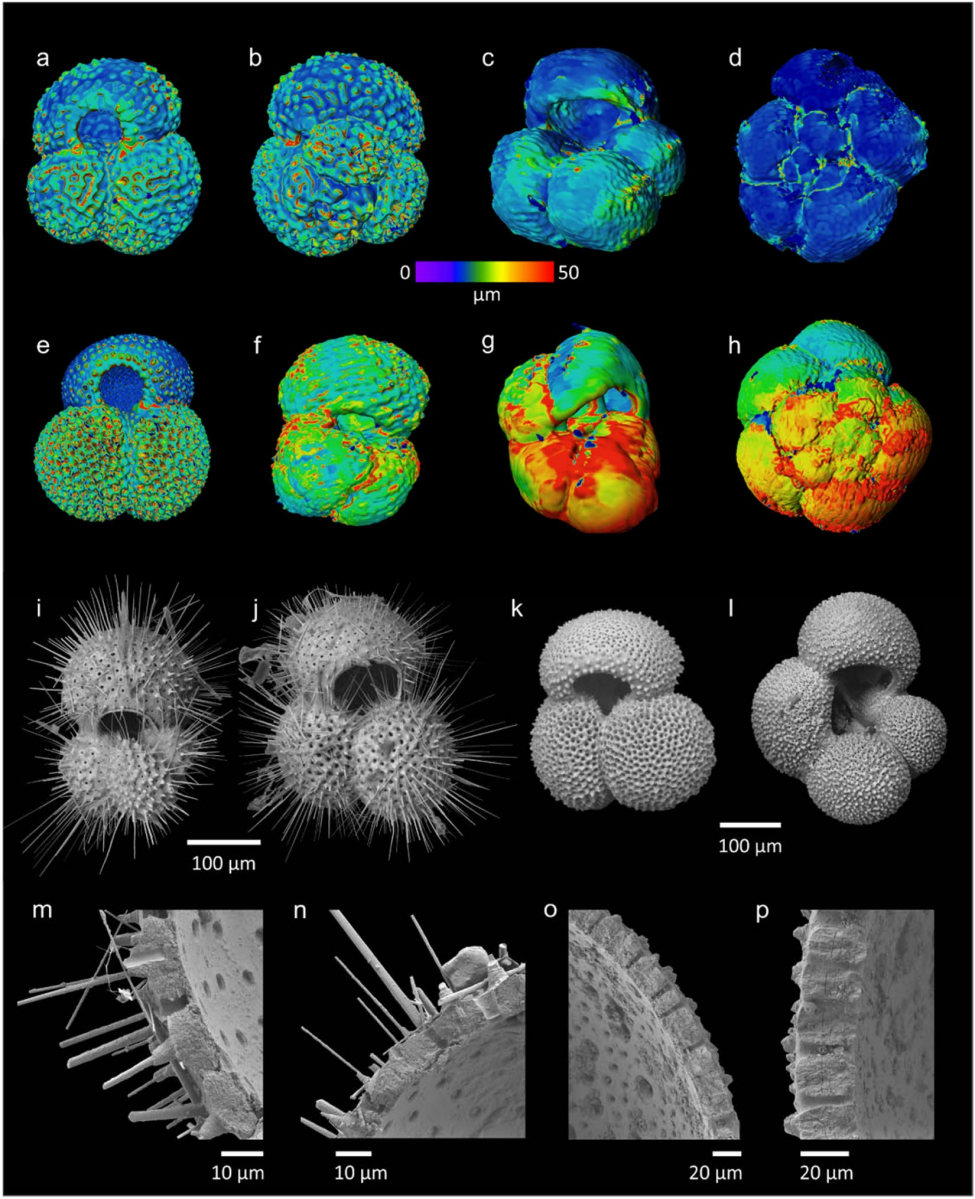
(**a**–**h**) Nano-CT scan of planktonic foraminifera specimens with colourmap of test thickness, warm colours indicating areas of relatively thicker shell; (**a**,**b**) *Globigerinoides ruber* (*Tara*), (**c**) *Globigerina bulloides* (*Tara*), (**d**) *Neogloboquadrina dutertrei* (*Tara*), (**e**) *G. ruber* (*Challenger*), (**f**) *Trilobatus trilobus* (*Challenger*), (**g**) *N. acostaensis* (*Challenger*), (**h**) *N. dutertrei* (*Challenger*); (**i**–**p**) SEM images of selected planktonic foraminifera specimens; (**i**) *T. trilobus* (*Tara*), (**j**) *G. ruber* (*Tara*), (**k**) *G. ruber* (*Challenger*), (**l**) *G. bulloides* (*Challenger*), (**m**,**n**) *G. ruber* test cracked to reveal wall texture (*Tara*), (**o**,**p**) *G. ruber* test cracked to reveal wall texture (*Challenger*).

**Figure 3:**
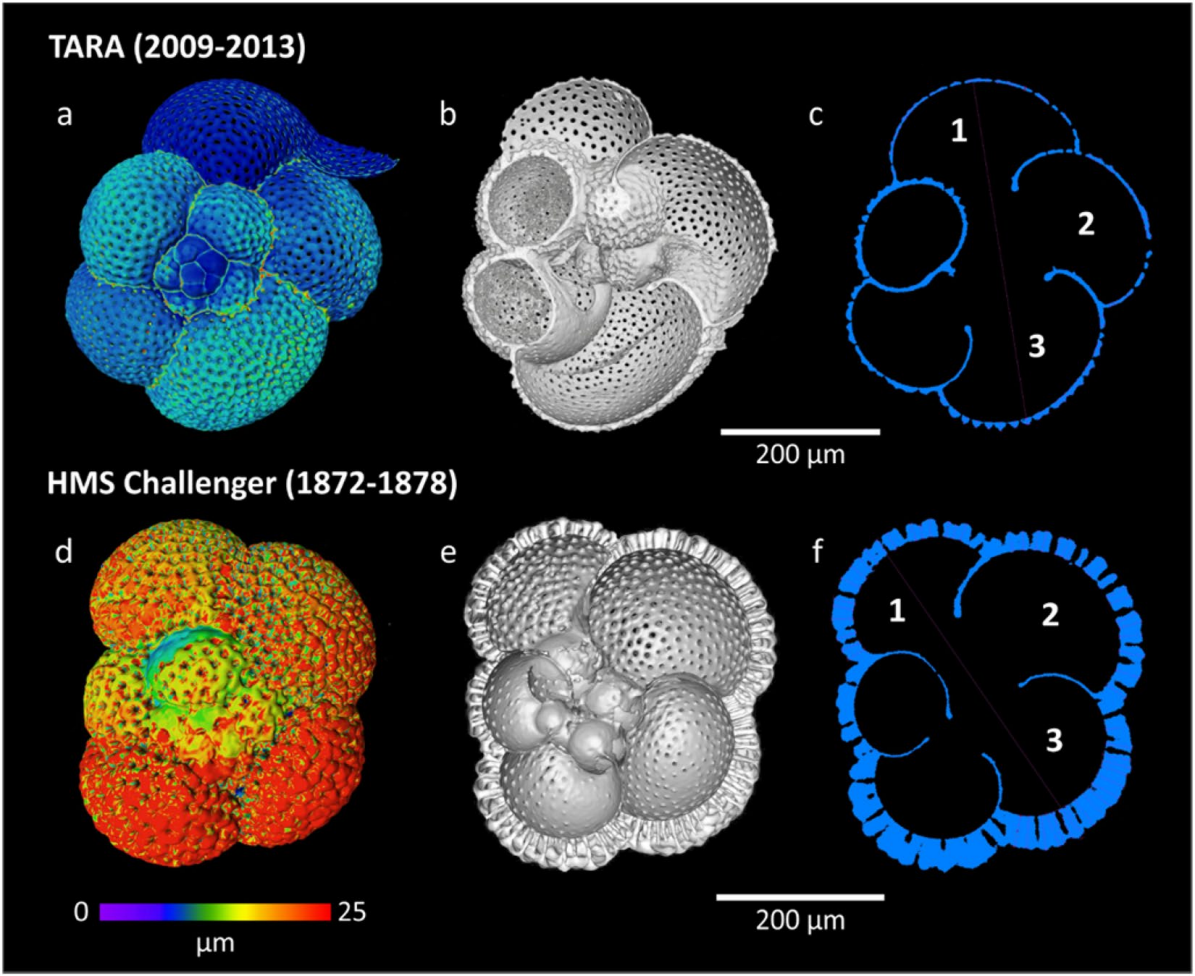
Nano-CT representative reconstructions and measurements for *Neogloboquadrina dutertrei* from *Tara* (**a**–**c**), and *Challenger* (**d**–**f**); 2a and (**d**) Colour map illustrating shell thickness, warm colours representing areas of relatively thicker test wall; 2b and (**e**) cross section through rendered foraminifera test; 2c and (**f**) slice through foraminifera test.

Here we test the utility of these collections for modern climate change studies by comparing specimens of planktonic foraminifera collected in tow nets from HMS *Challenger* Station 272 (1875) (Fig. 1) with *Tara* expedition (2011)^21^ stations 127 and 128, in the eastern Pacific Ocean; a region that is recognized as being particularly vulnerable to deoxygenation and surface warming in the wake of anthropogenic climate change^20^. Both *Tara* and HMS *Challenger* stations were sampled during September thus removing seasonal bias from our study. Tow samples that had been stored in ethanol immediately after collecting were analysed under light microscope and picked for planktonic foraminifera, with selected specimens imaged using a scanning electron microscope (SEM) to test for any visible signs of dissolution (Fig. 2). Of these, 24 specimens (12 historical and 12 modern) were then selected for imaging using X-ray computed tomography (CT). Nano-CT scanning provides an innovative technique for assessing shell properties, and offers the advantage of yielding many different parameters (wall thickness, chamber volumes etc.), per single scan. It also allows a comprehensive investigation into the entire shell, not just small sections, to display changes in shell thickness during the life cycle of the protozoan, whilst also taking into account areas of differing thickness due to shell structure morphology^22^ (Fig. 3).

**Figure 4:**
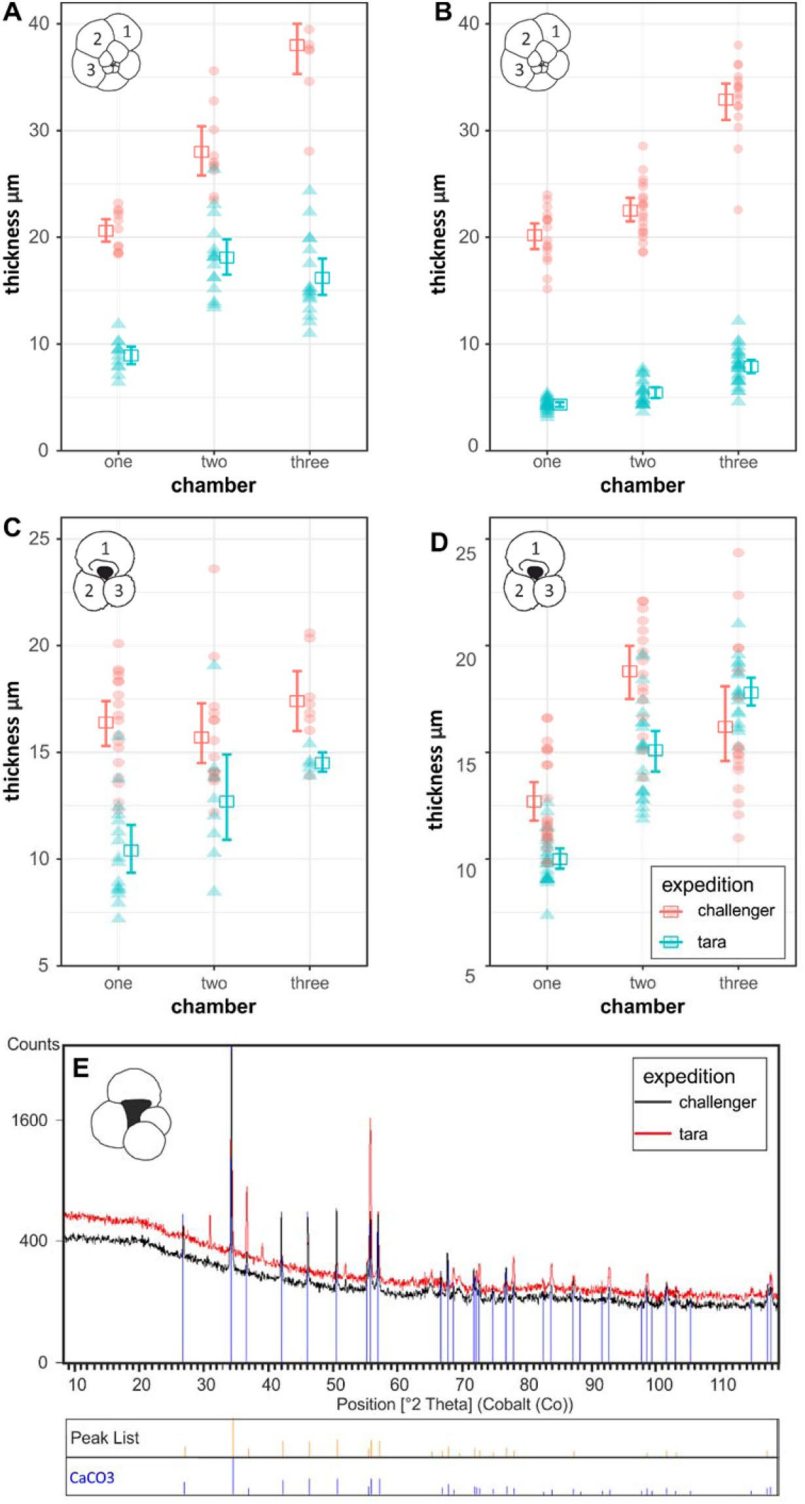
(**A**,**B**) Shell thickness measurements of *N. dutertrei*; (**C**,**D**) Shell thickness measurements of *G. ruber*. Pink circles represent HMS *Challenger* (1875) specimens, blue circles represent *Tara* (2011) specimens, squares represent the mean thickness for each chamber; (**E**) X-ray diffraction data for specimens of *G. bulloides*. Black line represents HMS *Challenger* data, red line represents *Tara* data.

Inspection of the outer surface of the shells using SEM revealed all selected specimens to be in good condition with no evidence of dissolution or recrystallisation^23,24^ (Fig. 2). A subsample of test calcite from *Globigerina bulloides* was further analysed using X-ray diffraction, which revealed no differences in CaCO_3_ peaks between the HMS *Challenger* and *Tara* specimens (Fig. 4).

The results of the Nano CT-scanning revealed that, without exception, all modern foraminifera specimens had measurably thinner shells than their historical counterparts (Figs. 3 and 4A–D). In addition, due to the extremely thin walls of several specimens from *Tara* data set, the initial voxel size of 2.4 μm was insufficient to properly render the data. A total of 4 specimens were scanned at sub-micron resolution (~0.7 um); 2 from HMS *Challenger* (1772–1876) samples and 2 from *Tara* Oceans (2009–2013) plankton tows. We analysed two species: *Globigerinoides ruber* and *Neogloboquadrina dutertrei*, and collected between 8 and 27 replicate measurements of test thickness per chamber, from the final 3 chambers of the test (Supplementary Table 1). A Shapiro Wilks normality test was performed on the chamber thickness data and an independent t-test assuming equal variance was subsequently selected to test whether the observed differences in shell thickness between preindustrial and modern specimens are significant.

Analysis of the higher resolution scans (~0.7 μm) revealed that specimens of *N. dutertrei* from both the historic and modern assemblages show the similar patterns of shell thickness, in that the walls of test chambers become progressively thinner towards the final chamber (Fig. 4A,B). The specimens analysed in the first scan (Fig. 4A) revealed a mean shell thickness of 28.60 ± 1.43 μm in the HMS *Challenger* specimen compared to 14.94 ± 0.73 μm recorded in the Tara specimen. The specimens of *N. dutertrei* analysed in the second scan (Fig. 4B) record similar results with a mean shell thickness of 24.99 ± 0.85 μm in the HMS *Challenger* specimen, and 5.96 ± 0.23 μm in the *Tara*, equating to a reduction in shell thickness of up to 76%.

Quantitative measurements of the test wall in specimens of *G. ruber* revealed mean test thickness of 16.34 ± 0.39 μm (Fig. 4C) and 15.5 5 ± 0.51 μm (Fig. 4D) for the HMS *Challenger* specimens and 11.74 ± 0.53 μm and 13.96 ± 0.45 μm in the *Tara* specimens. This equates to an overall reduction in shell thickness of up to 30%, however statistical analysis of the individual chambers in *G. ruber* revealed that the observed differences in test thickness of chamber 3, between the preindustrial and the modern were not significant. Over all this species records similar but less marked differences in the mean shell thickness compared to *N. dutertrei*. This may be due to differences in calcification between species.

## Discussion

Initial findings of the *Challenger* Revisited project have revealed a reduction in shell thickness of up to 76% in selected species of planktonic foraminifera over the last c. 140 years, which corresponds to a period of profound change in our oceans. Ocean acidification is not the only stressor faced by the world’s oceans in the coming decades and over the time period studied here. Rising temperatures and deoxygenation are also likely to have a substantial impact on marine ecosystems, and eastern boundary upwelling systems are likely to be strongly affected by all three stressors^20^. A recent study by Roemmich *et al*. (2012) comparing *Challenger* sea surface temperature measurements (1872–1876) to a more recent data set from the Argo Programme (2004–2010) revealed substantial warming of the modern upper ocean, with a warming signal that is global in its extent. We propose OA as the lead cause for the observed reduction in calcification of planktonic foraminifera as our results closely mirror results from laboratory studies and field observations from the Arabian sea^17^. However, It could be argued that rising temperatures, deoxygenation and OA are intrinsically linked and therefore these stressors should not be separated. More work is required to further explore the application of this method by increasing sample sizes and undertaking comparative studies in other ocean basins. Though the HMS *Challenger* samples only provide a snapshot of early industrial ocean conditions, the method has potential for global reconstructions. The “*Challenger* Revisited project” will be expanded beyond the Pacific Ocean, and the methodology can be applied to other historical expedition collections such as the *Terra Nova* [1911–1915] and *Discovery* [1920–1930s] when comparatory contemporary expedition materials are available.

Whilst all specimens analysed showed some reduction in shell thickness, the degree to which different species responded varied greatly. Specimens of *N. dutertrei*, a non-spinose, thermocline-dwelling planktic foraminifera that possesses intracellular chrysophyte alga^25,26^ revealed up to 76% reduction in shell thickness between the preindustrial and the modern, whereas *G. ruber* specimens display a far smaller decrease in shell thickness (~20%). *G. ruber* is a spinose multichambered species known to occupy the mixed layer of the ocean and hosts photosynthesising algal symbionts (dinoflagellates) which can alter the chemistry of the sea water immediately surrounding the shell and therefore enhance calcification^27,28^. Numerous studies have demonstrated that a variety of calcifying organisms respond negatively to decreasing ocean pH, such as coccolithophores, pteropods and corals^22,29,30^. However, certain photosynthesising organisms have been shown to benefit from higher availability of dissolved CO_2_^31^.

To further investigate all drivers for these differences in shell thickness, the effect of depth habitat on calcification ability is worth further study, as is how photosymbiont hosting foraminifera and also the types of symbionts may be more resilient to decreasing pH. Studies of the fossil record have shown that foraminifera, when under extreme stress, will shed their symbionts in the process of bleaching^32^.

In addition, the application of directed geochemical analyses have the potential to disentangle the multiple factors that could be driving the reductions in shell thicknesses shown here. Boron isotope analysis on comparable historic and modern planktonic foraminifera shells can provide accurate reconstructions of pH conditions at the time of shell development^33–35^, which in turn links the reduction of ocean carbonate ions as the driver for reduced shell calcification. There is therefore the potential to gain new insights into the rate of change taking place in the oceans, contributing greatly to our understanding of the sensitivity of global climate systems to CO_2_ forcing. Such insights are crucial for predicting the future climate and health of the oceans.

## Sample Information

Our preindustrial specimens were collected at HMS Challenger station 272 in the Southwest Pacific Ocean on the 8^th^ of September 1875 (3°48′0″S, 152°56′0″W), using a tow net “resembling long night-caps, of fine muslin or calico, and 10 to 16 inches in diameter at the mouth”^1^. In the HMS *Challenger* volumes the nets are reported as being towed at various depths, “even as far as 800 fathoms”^36^, however the ship’s station book does not record the depth at which the nets were towed at individual stations. Sea surface and bottom temperatures, the sounding, “nature of the sea floor” and species recovered are recorded. The expedition logs are publicly available at the library of the Natural History Museum, London.

A small sub-sample of the HMS *Challenger* material (50 g) was taken from the stores and gently washed using deionised water, the residues were dried in an oven at 40^o^ C. Specimens were selected from the 250–500 um size fraction for further analysis.

The *Tara* Oceans specimens came from station 127 (6°37′12.00″S, 152°34′4.80″W) and station 128 (0°28′8.04″S, 153°18′19.44″W) in the Pacific ocean and were collected between the 31^st^ of August and 5^th^ of September 2011 from the Deep Chlorophyll Maximum (DCM) layer (100 m)^21^. We analysed 10 ml aliquots from the 180–2000 um size fraction, samples were stored in a freezer in ethanol. Detailed accounts of the collection methodology can be found in Pesant *et al*. 2015.

Species identifications follow the existing understanding of modern foraminiferal taxonomy^37–39^. The primary classification is based on the chamber arrangement, wall structure and principally spinose or non-spinose ornamentation^40,41^.

## Methods

### Nano-CT scanning

Nano-CT scanning was carried out with a Zeiss Versa 520. Samples were analysed in pairs using a customised pine wood mount. Sample mount, X-ray source and detector geometry were kept constant throughout the first set of scans (Fig. 2). A scan resolution voxel (a 3D unit of space which varies in dimensions between CT reconstructions depending on scanning parameters) size of 2.4 µm^3^ was typically achieved using this set up in order to maximize the number of specimens that could be analysed in a single scan. During the second run of scans (Fig. 3) focusing on just 4 specimens (2 historic and 2 modern), X-ray source and detector were positioned to maximize resolution and a voxel size of <0.8 µm^3^ was achieved. Between 700–1000 individual X-ray absorption profiles of each mount were taken and combined to build a 3D rendering of the image using Avizo 9.2 software which was also used for image analysis.

The differential X-ray absorption of organic matter, pine wood, and calcium carbonate translates into different greyscale intensities in the reconstructed 3D image; therefore, it was possible to filter out pine wood and organic material from the analysed data for volume, wall thickness etc. Object volume, average thickness and surface area were determined using standard Volume Graphics analysis. In addition, dimension measurements were taken on 3D renderings of shells. For *N. dutertrei* and *G. ruber*, three dimensional measurements were taken, the width (at the widest point), the width at half shell length and length (Fig. 2a). Object volume, average thickness and surface area were determined using standard Volume Graphics analysis. For direct comparisons between shell thickness, weight and density of the old and new samples, a size filter was applied to the dataset and only individuals of the same length were used.

### X-ray diffraction

Specimens of *Globigerina bulloides* were picked for X-ray diffraction analysis as they were relatively abundant in both *Tara* and *Challenger* samples, between 5 to 10 shells were soaked in ethanol and gently ground in an agate mortar and deposited on a circular sapphire substrate. The XRD data were collected using an ENRAF-Nonius 590 diffractometer with a Tintel curved position sensitive detector (PSD). The angular linearity of the PSD was calibrated with silicone powder and silver behenate. This apparatus collects data from 2–120 °2θ continuously throughout the experiment. Cobalt Ka radiation was selected from the primary beam by a Germanium 111 crystal monochromator with the x-ray tube operating at 40 kV and 30 mA. Horizontal and vertical slits restricted the beam to a height of 0.14 mm and width of 5 mm. Data were collected with samples spinning continuously in the plane of the sample surface and with the sample surface at an angle of 4° to the incident beam.

### Detailed statistics

#### Neogloboquadrina dutertrei

Scan 1. In chamber 1 (the final chamber in the whorl), the preindustrial (*Challenger*) specimens recorded a mean shell thickness of 20.60 ± 0.56 μm, and a mean thickness of 8.93 ± 0.44 μm was recorded in the modern (*Tara*) specimen. A two sample t-test assuming equal variance was carried out. The t-statistic calculated was 16.593 and the two- tailed probability was 0.000. Therefore we can conclude there is a significant difference in shell thickness between the *Challenger* and *Tara* specimens.

In chamber 2, the preindustrial (*Challenger*) specimens recorded a mean shell thickness of 28.02 ± 1.2 μm, and a mean thickness of 18.12 ± 0.89 μm was recorded in the modern (*Tara*) specimen. A two sample t-test assuming equal variance was carried out. The t-statistic calculated was 6.71 and the two- tailed probability was 0.000. Therefore we can conclude there is a significant difference in shell thickness between the *Challenger* and *Tara* specimens.

In chamber 3, the preindustrial (*Challenger*) specimens recorded a mean shell thickness of 37.98 ± 1.30 μm, and a mean thickness of 16.19 ± 0.90 μm was recorded in the modern (*Tara*) specimen. A Mann Whitney U test showed that there was a significant difference (U = 170, p = 0.000) between the preindustrial and modern specimens.

Scan 2. In chamber 1 (the final chamber in the whorl), the preindustrial (*Challenger*) specimens recorded a mean shell thickness of 20.16 ± 0.65 μm, and a mean thickness of 4.33 ± 0.17 μm was recorded in the modern (*Tara*) specimen. A two sample t-test assuming unequal variance was carried out. The t-statistic calculated was 24.142 and the two- tailed probability was 0.000. Therefore we can conclude there is a significant difference in shell thickness between the *Challenger* and *Tara* specimens.

In chamber 2, the preindustrial (*Challenger*) specimens recorded a mean shell thickness of 22.55 ± 0.59 μm, and a mean thickness of 5.44 ± 0.27 μm was recorded in the modern (*Tara*) specimen. A two sample t-test assuming unequal variance was carried out. The t-statistic calculated was 26.521 and the two- tailed probability was 0.000. Therefore we can conclude there is a significant difference in shell thickness between the *Challenger* and *Tara* specimens.

In chamber 3, the preindustrial (*Challenger*) specimens recorded a mean shell thickness of 32.87 ± 0.90 μm, and a mean thickness of 7.89 ± 0.33 μm was recorded in the modern (*Tara*) specimen. A Mann Whitney U test showed that there was a significant difference (U = 432, p = 0.000) between the preindustrial and modern specimens.

#### Globigerinoides ruber

Scan 1 In chamber 1 (the final chamber in the whorl), the preindustrial (*Challenger*) specimens recorded a mean shell thickness of 16.60 ± 0.59 μm, and a mean thickness of 10.38 ± 0.59 μm was recorded in the modern (*Tara*) specimen. A two sample t-test assuming equal variance was carried out. The t-statistic calculated was -7.459 and the two- tailed probability was 0.000. Therefore we can conclude there is a significant difference in shell thickness between the *Challenger* and *Tara* specimens.

In chamber 2, the preindustrial (*Challenger*) specimens recorded a mean shell thickness of 15.74 ± 0.74 μm, and a mean thickness of 12.74 ± 1.12 μm was recorded in the modern (*Tara*) specimen A Mann Whitney U test showed that there was a not a significant difference (U = 95.5, p = 0.000) between the preindustrial and modern specimens.

In chamber 3, the preindustrial (*Challenger*) specimens recorded a mean shell thickness of 17.40 ± 0.78 μm, and a mean thickness of 14.52 ± 0.45 μm was recorded in the modern (*Tara*) specimen. However insufficient replicate measurements were collected (<10) to perform statistical analysis of the data.

Scan 2. In chamber 1, the preindustrial (*Challenger*) specimens recorded a mean shell thickness of 12.71 ± 0.25 μm, and a mean thickness of 10.01 ± 0.47 μm was recorded in the modern (*Tara*) specimen. A Mann Whitney U test showed that there was a significant difference (U = 58.5, p = 0.000) between the preindustrial and modern specimens.

In chamber 2, the preindustrial (*Challenger*) specimens recorded a mean shell thickness of 18.77 ± 0.64 μm, and a mean thickness of 15.06 ± 0.48 μm was recorded in the modern (*Tara*) specimen. A two sample t-test assuming equal variance was carried out. The t-statistic calculated was 4.749 and the two- tailed probability was 0.000. Therefore we can conclude there is a significant difference in shell thickness between the *Challenger* and *Tara* specimens.

Statistical analysis of the measurements taken on chamber 3 suggest that any observed difference in shell thickness is not significant.

## Supplementary information

Dataset 1.
